# Auditory and Visual Interhemispheric Communication in Musicians and Non-Musicians

**DOI:** 10.1371/journal.pone.0084446

**Published:** 2013-12-27

**Authors:** Rebecca Woelfle, Jessica A. Grahn

**Affiliations:** 1 Department of Biology, University of Western Ontario, London, Ontario, Canada; 2 Brain and Mind Institute, University of Western Ontario, London, Ontario, Canada; 3 Department of Psychology, University of Western Ontario, London, Ontario, Canada; UNLV, United States of America

## Abstract

The corpus callosum (CC) is a brain structure composed of axon fibres linking the right and left hemispheres. Musical training is associated with larger midsagittal cross-sectional area of the CC, suggesting that interhemispheric communication may be faster in musicians. Here we compared interhemispheric transmission times (ITTs) for musicians and non-musicians. ITT was measured by comparing simple reaction times to stimuli presented to the same hemisphere that controlled a button-press response (uncrossed reaction time), or to the contralateral hemisphere (crossed reaction time). Both visual and auditory stimuli were tested. We predicted that the crossed-uncrossed difference (CUD) for musicians would be smaller than for non-musicians as a result of faster interhemispheric transfer times. We did not expect a difference in CUDs between the visual and auditory modalities for either musicians or non-musicians, as previous work indicates that interhemispheric transfer may happen through the genu of the CC, which contains motor fibres rather than sensory fibres. There were no significant differences in CUDs between musicians and non-musicians. However, auditory CUDs were significantly smaller than visual CUDs. Although this auditory-visual difference was larger in musicians than non-musicians, the interaction between modality and musical training was not significant. Therefore, although musical training does not significantly affect ITT, the crossing of auditory information between hemispheres appears to be faster than visual information, perhaps because subcortical pathways play a greater role for auditory interhemispheric transfer.

## Introduction

Music is a feature of human cultures around the world, and the encoding and production of music in the brain is a complex process that involves the coordination of multiple sensory modalities and motor circuits [1], [2]. Musical experts, who have engaged in years of musical training, master high degrees of motor synchronization and temporal accuracy on the basis of visual and auditory cues. A key element of brain processing that allows optimal musical performance is the interhemispheric transfer of information. Most interhemispheric transfer of information channels through the CC, composed of axonal fibre bundles connecting the right and left hemispheres [3]. In order for a musician to play an instrument effectively, the CC must relay sensory and motor information quickly between hemispheres.

There has been abundant research on interhemispheric communication, with Poffenberger [4] among first to investigate interhemispheric transmission times (ITTs). Poffenberger calculated the ITT of visual stimuli in humans, using reaction time (RT) tests. The Poffenberger paradigm involves presenting visual stimuli to the right or left visual field, and participants respond to each stimulus with their right or left hand [5]. In the uncrossed condition, visual sensory information is presented to the same hemisphere that controls the motor response (ipsilateral presentation). In the crossed condition, visual sensory information is presented to the opposite hemisphere requiring information to travel across hemispheres to trigger a motor response (contralateral presentation). Thus, ipsilateral presentation (e.g., visual stimulus in the right visual field with a right hand response) requires no interhemispheric transfer of information, whereas contralateral presentation does (e.g., visual stimulus in the right visual field with a left hand response). In Poffenberger's experiment, ITT was determined by subtracting the reaction time of the uncrossed condition from the reaction time of the crossed condition. The difference between crossed and uncrossed reaction times (also known as the crossed-uncrossed difference, or CUD) can be used as a measurement of ITT [6]. The key finding was that uncrossed reaction times were shorter than crossed reaction times [4], and further studies have estimated mean ITT to be between 3–6 ms (for a review, see [6]).

Although most previous studies have examined ITT using only visual stimuli (for a review, see [6]), a few experiments have used auditory stimuli. Bjorklund and Lian [7] measured CUDs from an auditory two-choice reaction time test. During the test, a tone was presented in the left or right ear, and participants responded by choosing left or right ‘target buttons'. They chose the left target button with their left hand, and right target button with their right hand. The reaction time for uncrossed conditions was shorter than for crossed conditions (249 ms and 297 ms, respectively). However, two-choice reaction time tests are different from the simple reaction time tests used in previous visual studies, thus the results are not directly comparable. Bohr et al. [8] used an auditory simple reaction time test, and found that uncrossed RTs were 7.4 ms and 1.5 ms faster than crossed RTs for right and left hand responses, respectively. Therefore, the range of CUDs for auditory and visual stimuli appears similar when using simple RT tests.

A mechanism for visuomotor communication in the crossed and uncrossed conditions is described by Harvey [9]. In the crossed condition, synaptic transmission causes a delay in the transfer of information between hemispheres. In the uncrossed condition, a target in the right visual field is in the receptive field of the right nasal retina, and the left hemisphere processes the stimulus. Sensory information travels directly from the left visual area to the left motor cortex, which controls the right hand. In contrast, for the crossed condition, a target in the left visual field is in the receptive field of the right temporal retina and the right hemisphere processes the stimulus. However, visual sensory information must then cross the CC to the left motor area to trigger a response from the right hand. The pathway involving interhemispheric crossing is therefore less direct and requires additional time for synaptic transmission, resulting in a longer reaction time than uncrossed conditions [9].

Presently, there are no studies that have examined non-human animal CUDs. However, one study measured electrophysiological response times of the auditory cortex in the right and left hemispheres of anesthetized cats [10]. It was found that auditory ipsilateral response times were shorter than auditory contralateral response times. This suggests a similar interhemispheric transfer process exists for auditory stimuli, and that if the auditory stimulus is opposite to the responding hand, there must be interhemispheric transfer to produce a response.

Some human studies have used electroencephalography (EEG) techniques to examine interhemispheric communication. To measure ITT, Westerheusen et al. [11] examined responses to visual stimuli using event-related potentials. Regions of the posterior CC that contained a higher density of myelin and/or membrane had faster ITTs compared to other callosal regions. Additionally, Patston and colleagues [12] used EEG to measure ITT in musicians and non-musicians performing a simple reaction time test to visual stimuli. Non-musicians showed a faster ITT than musicians when information traveled in the right-to left direction, and both non-musicians and musicians had similar ITTs in the left-to-right direction. However, musicians showed a more equilateral transfer of information across the CC. Patston et al. [12] proposed that enhanced bilateral neural connectivity is a result of increased bimanual training in musicians.

As mentioned previously, the site of interhemispheric transfer is thought by many to be the CC [13], [14], [15]. The CC is generally divided anteriorly to posteriorly into the rostrum, genu, body, isthmus, and splenium [16]. The complete maturation of this brain structure occurs by the age of ten, and it is one of the last sets of fibre tracts to be myelinated [17], [18]. During childhood, the CC undergoes extensive development in order for humans to attain the adult level of bimanual coordination [14].

Many studies have shown a relationship between callosal size and musical training during callosal development [13], [19]. Using magnetic resonance imaging (MRI), Schlaug et al. [13] examined differences in callosal size of early-trained musicians (i.e., before the age of 7), late-trained musicians, and those with no musical training (non-musicians). Early-trained musicians had a larger mid-sagittal anterior CC than late-trained and non-musicians. Schlaug et al. [13] suggest that the larger anterior CC may come from a greater number of fibres crossing between hemispheres, however, there may be more myelination and increases in the diameter of the callosal axons. Therefore, differences in callosal size for musicians may be due to either a greater number of axonal fibres or greater myelination and axon diameter. If it is the latter, then we would expect smaller CUDs for musicians because greater myelination involves more rapid conduction of action potentials and faster transmission [13].

Musicians and nonmusicians also show differences in the brain stem and auditory cortex structure and physiology, which could influence ITT. Musicians show earlier and larger brainstem responses to music and speech stimuli than nonmusicians [20]. In addition, musicians show more robust and faithful brainstem encoding of speech stimuli [21]. Structurally, musicians show larger grey matter volume in primary auditory processing areas such as Heschl's gyrus [22], and structural volume correlates with the amplitude of electrophysiological responses to sound. Therefore, musicians appear to have enhanced processing of auditory information. The subcortical components of this circuitry may allow for faster processing and shorter ITTs.

Previous research shows that visual and auditory sensory fibres and motor fibres have different transcallosal pathways. Visual fibers cross at the posterior third of the CC [23], whereas auditory fibres cross at the posterior portion of trunk and anterior splenium [24]. In contrast, the crossing of motor fibres is proposed to be through the rostral body and anterior midbody [16]. Therefore, the site of interhemispheric transfer could be in sensory or in motor areas and may vary depending on sensory modality. However, Tettemanti et al. [15] found that the genu of the CC is the site of interhemispheric transfer in the Poffenberger paradigm, and that crossing of visual and auditory information happens through motor areas, instead of sensory-specific areas. They used functional magnetic resonance imaging (fMRI) to examine activated cortical regions, during a simple visual reaction time test (ITT was calculated as in the Poffenberger paradigm). Results from the fMRI show that, in the crossed condition, the genu was activated. Although it is only recently that white matter activation has been reported in fMRI, several studies have shown that white matter activation is measureable, particularly in regions of the corpus callosum that have been implicated in interhemispheric transfer [25], [26].

The site of interhemispheric transfer for both modalities may be the genu of the CC [15] and therefore any structural changes that enhance transfer would apply to both modalities equally. However, a study by Iacoboni and Zaidel [27] suggests that interhemispheric transfer of auditory information may rely less on the CC, and more on subcortical pathways. Testing of a commissurotomized patient revealed larger CUDs than normal (consistent with the corpus callosum being important for rapid interhemispheric transfer of visual information), but auditory CUDs that were small (less than 5 ms). This may mean that auditory interhemispheric information transfer does not depend on the corpus callosum. Alternatively, it is possible that the patient had developed compensatory connections, and that their auditory CUDs were not representative of those that would be observed in the healthy population.

In the current study, the objective was to determine if musical training affects interhemispheric transmission of visual and auditory sensory information. We measured CUDs for both visual and auditory modalities as an indication of ITT, using a simple reaction time paradigm. We used a within-subjects design to compare auditory and visual CUDs in the same subjects.

We hypothesize that the site of information transfer for both auditory and visual information is the genu of the corpus callosum, thus auditory and visual CUDs should be similar [15]. Because musical training affects callosal development, we predict that musicians will have faster ITTs (smaller CUDs) than non-musicians in both modalities. Alternative patterns of results could emerge, however. For example, if only auditory CUDs, but not visual CUDs, are shorter in musicians than in non-musicians, this would suggest that the sites of information transfer differ for auditory and visual modalities, and musicians have faster ITTs for auditory information (which may or may not be mediated by the corpus callosum). Alternatively, if musical training has no effect on information transfer, either through the corpus callosum or subcortical structures, then CUDs for musicians and non-musicians will be the same.

## Materials and Methods

### Participants

Data were acquired from 60 participants (43 females, 17 males; mean age =  19.9 years, *SD* =  2.3), with 2 left-handers and 58 right-handers. Out of the 60 participants, 30 were non-musicians (less than 1 year of musical training of any kind) and 30 were musicians. All musicians had at least five or more years of formal musical training in one or more of various wind, stringed, percussion, or keyboard instruments. No musicians were solely vocalists. The age at which musicians started musical training ranged from 3–13 years (Mean = 6.0, *SD* = 2.7).

### Ethics Statement

The Psychology Research Ethics Board (PREB) at the University of Western Ontario approved the study, and participants were recruited from the Psychology Research Participation Pool. They were given 1.5 research credits for one and a half hours of participation. Written informed consent was obtained from all participants.

### Experimental Design

The design was based on the Poffenberger paradigm, and a unimanual simple reaction time test was used [4]. Testing was conducted on a PC desktop, and E-prime (Version 2.0) software [28] was used for stimulus presentation and recording of reaction times. Visual and auditory conditions were counterbalanced across participants. There was approximately a 1–2 minute break between each block and between the two modalities. For the visual test, participants practiced 2 blocks of 20 trials each prior to testing. For the test phase, there were 20 blocks, each containing 20 trials, for a total of 400 trials. Due to a technical error, three subjects (two non-musicians and one musician) were tested with 25 blocks (500 total trials). All data from these participants were included in the statistical analysis.

Participants used one hand to respond throughout an entire block, alternating the use of their left and right hand on each block. The hand started with (right or left) and modality type (visual or auditory) were counterbalanced across participants. Participants were instructed which hand to use (right/left) before each block. A black fixation cross was present in the middle of the computer screen, and participants were asked to maintain fixation throughout the task. A webcam was present (although not turned on) to encourage participants to keep their eyes fixated to the center of the screen. On each trial, a circle appeared on either the left or right side of the cross at a random time between 1.5 and 2.5 seconds after the previous response. Participants were instructed to press the SPACE bar as quickly as possible after the circle appeared. The side of stimulus presentation (right or left) was randomized from trial to trial.

For the auditory CUD test, participants had the same number of blocks and trials as the visual CUD test. Three participants (two non-musicians and one musician) were tested with 25 blocks (500 total trials) instead of 20 blocks. The extra trials were included in the statistical analysis. Participants used one hand to respond throughout an entire block, alternating the use of their left or right hand on each block. Auditory stimuli, in the form of beeps, were presented through a pair of Sennheiser HD 280 headphones. Each beep was a sine tone played for 0.05s, and had a frequency of 452 Hz. On each trial, a beep was presented at a random time between 1.5 and 2.5 seconds after the previous response, and participants had 1 second to respond. Participants were instructed to press the SPACE bar as quickly as possible after each beep. The side of stimulus presentation (left ear/right ear) was randomized from trial to trial. After completing the visual and auditory CUD tests, participants completed a demographic questionnaire about their musical training background.

### Statistical Analysis

Reaction times under 100 ms were considered false anticipatory responses, and were excluded. In addition, the longest 10% of reaction times were trimmed in each condition (visual crossed, visual uncrossed, auditory crossed, auditory uncrossed) for each participant to reduce the influence of outliers that could result from lapses in attention [29]. The crossed-uncrossed difference (CUD) was calculated by subtracting the mean reaction time of the uncrossed condition from the mean reaction time of the crossed condition in each modality. CUDs were analyzed with a 2 (visual/auditory) x 2 (musician/non-musician) mixed measures analysis of variance (ANOVA). Follow-up paired samples t-tests were conducted to compare differences between visual and auditory CUDs within each group. All data analysis was performed using SPSS [30] and Microsoft Excel [31] software. An α level of 0.05 was used for all statistical tests, and we performed two-sided tests.

## Results

Data from one participant were removed from analysis because of an abnormally high CUD in the visual reaction time test (M_CUD_ =  349.01 ms, >3 SD above the mean CUD for all participants). For the remaining 59 participants, 2566 (12%) of auditory responses were excluded, leaving 10583 crossed and 10636 uncrossed auditory responses in the analysis. For the visual modality, 2841 (14%) of responses were excluded, leaving 10324 crossed and 10444 uncrossed responses in the analysis.

**Figure 1:**
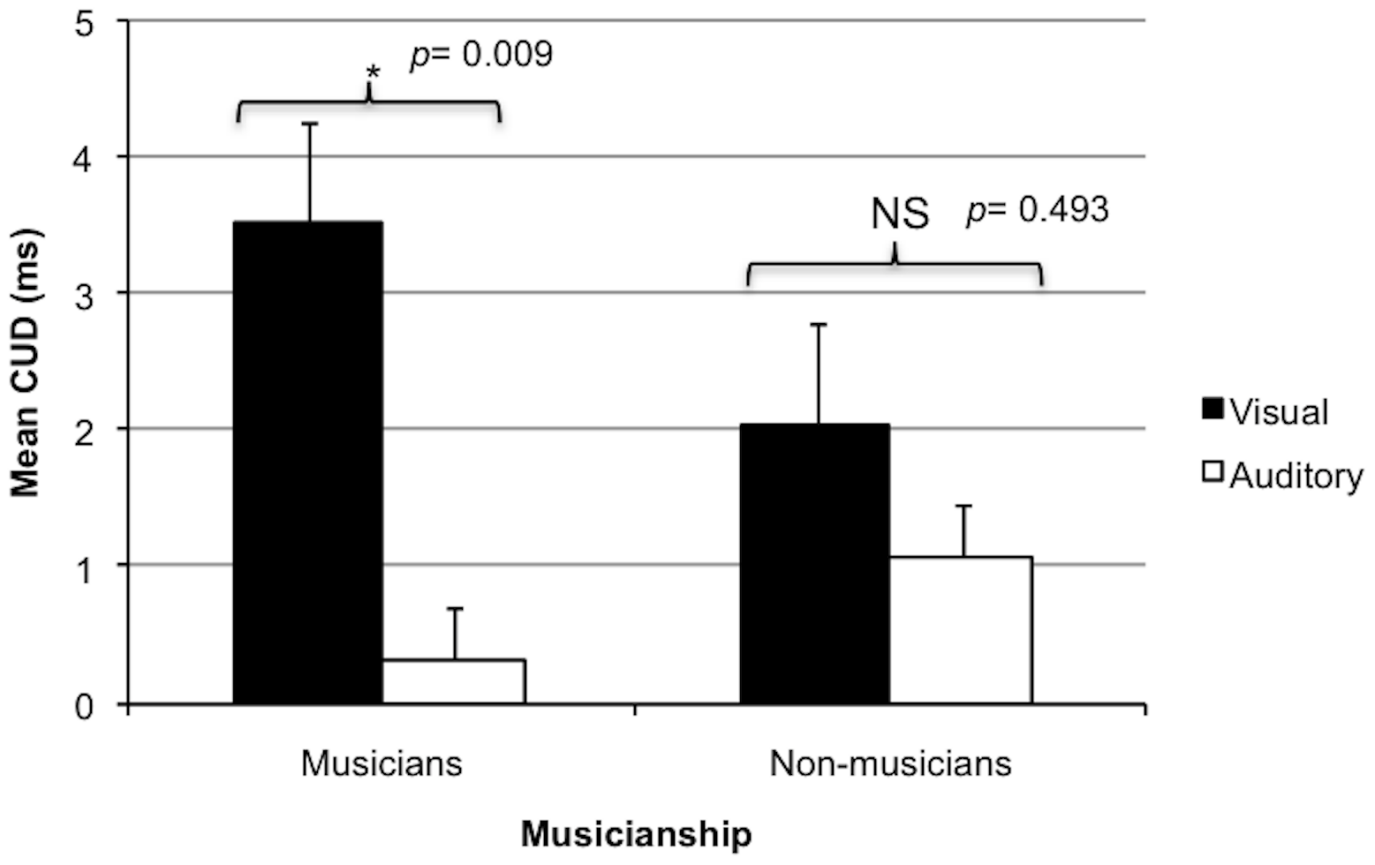
Crossed-uncrossed reaction time differences of visual and auditory modalities in musicians and non-musicians. CUDs in milliseconds (ms) were calculated by subtracting the mean reaction times of the uncrossed condition from the mean reaction times of the crossed condition. **The interaction between Musicianship and Modality is not significant (** ***p*** **=  0.22).**

For the auditory modality, crossed and uncrossed absolute reaction times were 278.4 ms and 277.7 ms, respectively. For the visual modality, crossed and uncrossed absolute reaction times were 291.9 and 289.1 ms, respectively. Auditory CUDs were smaller than visual CUDs as confirmed by a significant main effect of modality (*F_1, 57_* = 5.33, *p* =  0.025, *d* =  0.085, auditory: *M* = 0.704, *SD = *4.95; visual: *M* =  2.76, *SD* =  4.36) There was no significant effect of musical training (*F_1,57_* =  0.192, *p* =  0.663, musicians: *M* =  1.92 ms, *SD* =  4.58 ms; non-musicians: *M* =  1.56 ms, *SD* =  4.96 ms). The interaction between musical training and modality was also not significant (Figure 1; *F_1, 57_* = 1.52, *p* =  0.222). For musicians, the range of CUDs in the visual modality was −5.3 to 10.5 ms, and in the auditory modality was −7.9 to 10.8 ms. For non-musicians, the range of CUDs in the visual modality was −4.8 to 13.3 ms and in the auditory modality was −11.9 to 14.6 ms. Results from one-tailed one sample t-tests show that auditory CUDs were not significantly greater than zero (p =  0.279), and that visual CUDs were significantly greater than zero (p< 0.001).

Follow-up paired samples t-tests indicated that for musicians, auditory CUDs were significantly smaller than visual CUDs, (3.51±4.34 ms versus 0.32±4.30 ms; *t_28_* =  2.82, *p* = 0.009). For non-musicians, auditory and visual CUDs were not significantly different (2.04±4.32 ms versus 1.07±5.56 ms; *t_29_* = 0.694, *p* = 0.493).

An analysis of only the first half of trials shows a similar pattern of results compared to the full experiment. We found a significant main effect of modality (*F_1, 57_* = 4.97, *p* =  0.030, *d* =  0.080, auditory: *M* = −0.13, *SD* =  7.06; visual: *M* = 2.34, *SD* = 5.95), no significant interaction between modality and musicianship (*F_1, 57_* = 0.420, p =  0.520), and no significant effect of musical training (*F_1,57_* =  0.071, p =  0.791, musicians: *M* = 0.927 ms, *SD* = 6.69 ms; non-musicians: *M* =  1.27 ms, *SD* =  6.38 ms). Musicians had significantly smaller auditory than visual CUDs (−0.673±6.81 ms versus 2.53±6.57 ms; *t_28_* =  2.24, *p* =  0.033), and non-musicians had no significant differences between auditory and visual CUDs (0.393±7.37 ms versus 2.15±5.39 ms; *t_29_* = 1.04, *p* =  0.309). Thus, the pattern of results for the first half of the experiment is similar to that observed for the entire experiment.

To compare our results with previously reported differences in right-to-left and left-to-right information transfer for musicians and non-musicians [12], we used paired t-tests to compare CUDs separately for right-to-left transfer (stimulus presented to right hemisphere, response controlled by left hemisphere) and left-to-right transfer (stimulus presented to left hemisphere, response controlled by right hemisphere) in musicians and non-musicians. For both musicians and nonmusicians, right-to-left visual CUDs were not significantly different from left-to-right visual CUDs (musicians: 3.76±8.44 ms versus -0.523±7.31 ms, *t_28_ = * 1.72, *p* =  0.096; nonmusicians: 2.34±6.25 ms versus 0.960±6.81 ms, *t_29_ = *0.710, *p* = 0.484). For nonmusicians, there was a significant difference between right-to-left and left-to-right auditory CUDs (−1.69±6.39 ms versus 2.34±6.95 ms, *t_29_* = −2.09, *p* =  0.046), but not for musicians (−0.518±7.41 ms versus 1.34±6.46 ms, *t_28_* =  −0.863, *p* =  0.395).

## Discussion

The current experiment tested the effects of modality and musical training on interhemispheric transmission times. We predicted that visual and auditory CUDs would be similar. Overall, however, visual CUDs were significantly larger than auditory CUDs. This difference appeared to be driven by musicians: the interaction between modality and group was not significant, but musicians showed significantly larger visual than auditory CUDs, whereas non-musicians showed similar auditory and visual CUDs (see Figure 1). It is theoretically possible that musicians have developed a greater number of CC fibres connecting auditory areas at the cost of visual processing, but this possibility needs to be tested further using other techniques before any conclusions can be reached.

As mentioned previously, the genu of the CC has been proposed to be the site of interhemispheric transfer of information [15], in part based on fMRI activation observed in the genu during interhemispheric transfer. However, callosal activation may be measureable with fMRI only at anterior callosal sites [15], [32], thus activation at more posterior sites may have been present but not observed. Weber et al. [33] have hypothesized that interhemispheric transfer of sensory information may occur in parallel at multiple sites in a “horse-race” fashion, as several cortical areas are activated during ITT. Furthermore, Iacoboni and Zaidel [34] found that transfer of information occurs in parallel at the posterior parietal, prefrontal, and premotor areas during a simple reaction time test. Therefore, it is possible that the modality difference between auditory and visual CUDs results from differences in crossover sites.

This is the first study to directly compare auditory and visual CUDs in the same healthy participants. We found smaller auditory CUDs than visual CUDs, suggesting a difference in pathways for the two modalities. This is in accordance with work by Iacoboni and Zaidel [27] that suggests that the CC does not have the same role for auditory CUDs as visual CUDs. They obtained visual and auditory CUDs from a commissurotomized patient. The patient showed a small auditory CUD (4.25 ms) that was within the normal range, but a very large visual CUD (ranging from 25–45 ms). The smaller auditory CUDs relative to visual CUDs may indicate that subcortical transfer exists for the auditory modality but not the visual modality. However, it is possible that in healthy volunteers, who have intact callosal fibers, the transfer that dominates behavior occurs at cortical level, whereas in the case of complete commissurotomy, subcortical pathways are used to compensate and control behaviour [27]. The current study also found smaller auditory CUDs, Therefore, our results may be consistent with the possibility of subcortical transfer resulting in faster auditory than visual CUDs, even in healthy volunteers.

Subcortical circuits may play a role interhemispheric processing of auditory information. In the subcortical pathway for auditory processing, sound is transmitted from the inner hair cells of the cochlea to auditory nerve fibres connecting to the cochlear nucleus in the pons [35]. Then, auditory signals travel from the cochlear nuclei to both the contralateral and ipsilateral lateral lemniscus, which subsequently innervates the inferior colliculus in the midbrain [36]. There are also contralateral projections between lemniscal nuclei on each side, and from one inferior colliculus to the other [37]. The thalamus' medial geniculate nucleus receives afferent auditory input from the inferior colliculus, and then transmits auditory signals to Heschl's gyrus of the primary auditory cortex (site of auditory processing) [38]. Previous research suggests that musical training affects neural structures, including the CC, during childhood [13], and therefore it is plausible that musicians who began training when callosal development was still occurring (before age 7) might show faster interhemispheric transfer than non-musicians. As previously stated, Schlaug et al. [13] found that early-trained musicians had a larger mid-sagittal anterior CC than late-trained and non-musicians. A larger CC might be composed of axon fibres with larger diameter or greater myelination, thus reducing CUDs [39] through faster interhemispheric transmission time. However, it may be that the larger callosal size results from a greater number of axons [40], rather than larger diameter or more myelination. A greater number of axons would not necessarily cause faster interhemispheric transmission. Therefore, as we did not find smaller CUDs in musicians than non-musicians, our findings support that the cross-sectional CC size difference may result from a greater number of axons.

Past work has compared reaction times in musicians and non-musicians, but not compared crossed and uncrossed conditions. Brochard et al. [41] measured reaction times using both simple reaction time and choice reaction time tests. The simple reaction time involved a button-press response to visual stimuli, while the choice reaction time was a colour discrimination task. In both simple reaction time and choice reaction time tests, musicians had significantly faster reaction times than non-musicians. Brochard et al. [41] explains that musicians may perform better than non-musicians because they have better sensorimotor integration. Also, musicians may have an “over-learned ability” to associate a visual stimulus with a motor response, and more efficient mechanisms used in visual processing (e.g. honed through musical score reading) that allow them to have faster reaction times [42]. These results differ from the current study, as we did not find a significant difference in reaction times to visual stimuli when comparing musicians (291.3 ms) and non-musicians (289.8 ms). Our results may differ because our definition of “musician” was not as strictly defined as Brochard et al.'s [41]: our “musician” group had at least 5 or more years of musical training, whereas Brochard et al.'s [41] “musician” group had at least 8 years of musical training, could sight-read music, and practiced at least one musical instrument for more than four hours per week. Thus, the “musicians” in Brochard et al.'s [41] may have even more robust and efficient visuomotor circuits than “musicians” of the current study.

As mentioned previously, Patston et al. [12] compared ITTs between musicians and nonmusicians using EEG, instead of reaction times. To measure ITT, they subtracted the latency of the contralateral N1 from the latency of the ipsilateral N1 for both left visual field and right visual field conditions. They found faster ITTs in nonmusicians when information traveled in the right-to-left direction than in the left-to-right direction, and no significant directional differences in ITTs of musicians [12]. Using RTs to measure ITT, we did not find similar results, as nonmusicians' visual ITTs in the right-to-left direction were not significantly slower than in the left-to-right direction. We also found that nonmusicians had significantly faster auditory ITTs in the right-to-left than left-to-right direction, and no significant differences within musicians for auditory ITTs. Thus, our auditory, but not visual, differences are consistent with the EEG findings. Our visual modality results may differ from Patston et al.'s [12] because of the different methods used to assess ITT (i.e. RTs versus EEG). Using EEG may be a more direct method to measure ITT as latencies from both hemispheres are recorded simultaneously, and it may be easier to measure differences between right-to-left and left-to-right interhemispheric transfer [43].

The results of this study raise new questions for future research. Additional groups that could be examined are musicians who play instruments unimanually versus bimanually, such as brass, string, and piano players. Using the same unimanual simple reaction time test as the current experiment, bimanual instrument players may show more accurate responses and faster ITTs than unimanual players. Other groups that could be compared are musicians and athletes. It would be interesting to compare ITTs of these groups, as both involve quick, coordinated movements in response to visual and auditory stimuli. During a performance, however, musicians may focus more on auditory information, whereas athletes may focus more on visuospatial information.

### Conclusion

Overall, visual CUDs were significantly larger than auditory CUDs. A possible explanation for smaller auditory CUDs is that interhemispheric transfer of auditory information might not rely on the CC to the same degree as the transfer of visual information. Even though there was no significant interaction between musical training and modality, the differences between auditory and visual CUDs were only significant for musicians.
